# Putting Children’s Sleep Problems to Bed: Using Behavior Change Theory to Increase the Success of Children’s Sleep Education Programs and Contribute to Healthy Development

**DOI:** 10.3390/children3030011

**Published:** 2016-07-01

**Authors:** Sarah Blunden, Tessa Benveniste, Kirrilly Thompson

**Affiliations:** Appleton Institute, Central Queensland University, Adelaide South Australia 5000, Australia; t.benvensite@cqu.edu.au (T.B.) kirrilly.thompson@cqu.edu.au (K.T.)

**Keywords:** prevention, children’s sleep, behaviour theory, sleep education

## Abstract

Sleep is critical for the healthy development of children, yet most children simply don’t get enough. Whilst school based sleep education programs have been developed for parents and their children, they have had mixed success. We consider how existing school-based sleep education programs can be improved by applying a broader model to behaviour change theory. We find that the mixed success of school-based sleep education programs may be due to a plausible but misleading assumption that simply increasing information about the importance of sleep and the risks of insufficient and/or inefficient sleep, will necessarily result in improved sleep behaviours. We identify the potential benefits of using a more inclusive behavior change theory in the development of sleep education programs with a particular need for theories that incorporate the multiple biological, environmental and social impacts on children’s sleep. Bronfenbrenner’s Bioecological model is presented to illustrate how one such inclusive behavior change theory could significantly improve the success of sleep education programs and ultimately support the healthy development of children.

## 1. Introduction

### 1.1. The Detrimental Outcomes of Sleep Problems

The cost of sleep disorders in children has been estimated at $AUS 1.5 billion, for both primary and secondary consequences. Perhaps the significant value of addressing common sleep problems lies in the potential to reduce the burden of disease and mitigate the ongoing effects of sleep related issues in children and their families. Sleep is important for overall health and wellbeing, especially in children and adolescents [1]. A range of neuropsychological (cognition, attention and memory), and behavioural (aggression, hyperactivity) impairments have been recorded in relation to poor sleep [2]. Insufficient sleep quality and quantity have been associated with poor physical health including cardiovascular risks, compromised immune function, metabolic changes such as insulin resistance (a precursor to diabetes) and a greater likelihood of being obese [3,4]. Such health issues can persist into adulthood, causing further concern [5].

In addition to physical sequelae, poor sleep can have a detrimental impact on mental health [6,7]. More than 90% of depressed children and adolescents report problems with their sleep, with common complaints including insomnia, hypersomnia and daytime sleepiness [8]. In addition, a majority of youth presenting with primary complaints of insomnia also meet criteria for a mental health diagnosis [5,9]. Similarly, early sleep problems may independently predict the later development of emotional problems such as depression, anxiety, inattention and hyperactivity. A relationship between attribution styles, anxiety, and sleep problems is also apparent [10]. That is, sleep has been shown to affect emotions, with longer sleep duration being shown to predict prospectively higher self-esteem, with good sleep quality also showing higher levels of optimism and reduced stress [11,12]. Studies have shown that improving sleep quantity in young people has improved their mood. Although the interactions between sleep and physical/mental health in adolescents are more complex than we have space to explain here, it is not unreasonable to suggest that for some adolescents, poor sleep contributes to mood disorders and that improving sleep may have a positive effect on mental health and wellbeing.

### 1.2. Prevalence of Sleep Problems in Children and Adolescents

Depending on definitional criteria and type of measure, estimates of sleep disorders are between 20% and 41% of children presenting with sleep problems [13]. Clinically defined sleep disorders are classified as either physiological or behavioural. Physiological sleep disorders include obstructive sleep-disordered breathing, restless legs syndrome and delayed sleep phase syndrome (DSPS) (for reviews see [14,15,16,17]). Prevalence rates range between 2% and 3% of children and adolescents diagnosed with restless legs syndrome or obstructive sleep apnea [15], and up to 10% for delayed sleep phase syndrome, particularly in adolescents [18]. Whilst community awareness is important, amelioration of these sleep disorders can generally only be achieved with medical intervention.

Behavioural sleep disorders (non-physiological sleep disorders which are related to sleep habits and behaviours) are more common, with prevalence estimates between 30% and 40% [19]. Behavioural sleep disorders in young people are termed by the American Academy of Sleep Medicine (AASM) as Behavioural Insomnia of Childhood. Common behavioural sleep disorders include Insufficient Sleep Syndrome, Limit-Setting Sleep Disorder, Sleep-Onset Association Disorder and Inadequate Sleep Hygiene Disorder [20]. In many cases, the amelioration of these behavioural sleep disorders can be achieved without costly and invasive medical intervention by ameliorating the behaviours that surround sleep [21].

Sleep hygiene is the term used to describe sleep and bedtime related behaviours. Poor sleep hygiene, such as excessive use of electronic screens prior to sleep onset can be problematic even without reaching the clinical diagnosis of Inadequate Sleep Hygiene Disorder (AASM) [20]. Therefore, it is important for health educators, professionals and the wider community to be more aware of the value of sleep for child development and how to prevent behavioural sleep disorders from becoming clinical sleep disorders.

### 1.3. Treatment of Behavioural Sleep Disorders

Fortunately, behavioural sleep problems are amenable to change, often through modifications to sleep hygiene [22]. Treatment approaches often involve behavioural intervention, targeting either the parents or their child [19]. Interventions typically seek to improve children’s sleep overall by: increasing sleep duration, improving sleep quality, maintaining consistent sleep/wake schedules and minimising behaviours that are detrimental to sleep, such as excessive caffeine use or excessive physical activity prior to bedtime. Several studies have shown that the use of educational interventions to increase levels of awareness about sleep behaviour and sleep hygiene have been successful in ameliorating sleep disturbance, sleep wake schedules [23]. The expectation is that sleep education improves sleep and subsequently the secondary outcomes of poor sleep described above. But does it? And if not, why not?

## 2. Is Formal Sleep Education the Answer?

Despite the detrimental impacts of poor sleep and the numerous medical and behavioural interventions, health professionals and the general public are relatively uninformed of the high prevalence of sleep problems in children and adolescents [24]. This suggests a need to increase awareness and knowledge of sleep problems and their consequences, from ‘top-down’ as well as ‘bottom-up’ influences.

First, it is necessary to increase knowledge from ‘top-down’, amongst health professionals. Although sleep education for this group would focus on medical sleep disorders, many behavioural sleep disorders present co-morbidly with medically based sleep disorders (such as depression, obesity and reduced sleep duration) [25]. As such, sleep training and education would be valuable during medical training. In addition, health professionals working with children may want to consider basic screening for sleep problems. This can provide insight where there are concerns about a child’s daytime functioning [19] especially given the significant relationship between poor sleep at night and behavioural deficits during the day. But sleep education in medical curricula is not systematic and in most cases is limited to a few hours over the course of medical training [26].

Second, awareness of sleep problems should be raised from the ‘bottom-up’, amongst the general public. Several school-based sleep education programs have been developed, to increase general awareness and disseminate knowledge about healthy sleep behaviour practices through the school in the expectation that this would disseminate at a broad community level [27,28]. Of the school based sleep education studies that have evaluated sleep knowledge, most have seen significant increases in sleep knowledge, with some studies reporting a 94% increase post-program [29,30,31,32]. Yet this knowledge rarely equates to behavior change [27].

**Figure 1:**
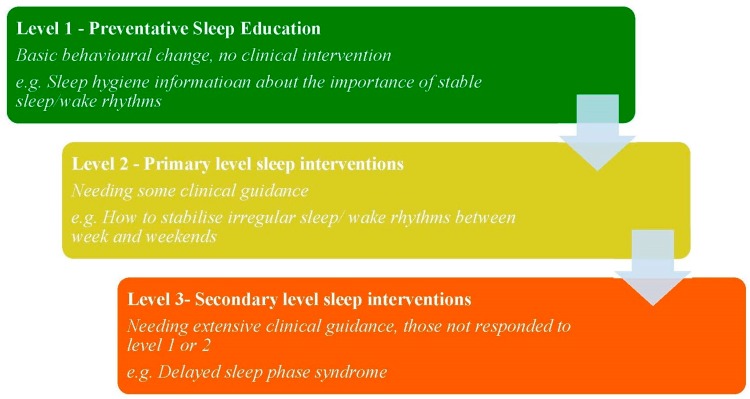
Proposed adaptation of the stepped care model for sleep education.

The delivery of sleep education at a population level is a principal component of the preventative health care model that has previously been adapted to sleep health [33]. This model (see Figure 1) suggests that delivery of early, evidence based sleep information is likely to prevent sleep problems from emerging and from developing into more serious and consequential problems in the future.

As shown in Figure 1, certain sleep disorders could be improved or even eliminated through information-based interventions alone, negating the need for clinical intervention. For example, in level 1, sleep hygiene information (described as helpful sleep promoting behaviours) could include warnings of the poor outcomes related to catch up sleeps on weekend which result in a significant difference between bed and wake times between weeks and weekends. These variations in the stability of sleep/wake schedules are a risk factor for DSPS. In fact, Lack et al. [34] describe how sleeping in on weekends can exacerbate biological sleep disorders such as DSPS and that it is better to catch up sleep through napping instead to prevent this variation in sleep wake stability from developing into the more severe problem of DSPS. So early intervention for sleep disorders requires at least an element of education and can be beneficial.

Yet the premise of the stepped care model is that dissemination of information will result in behavior change and whilst some school based sleep education programs have been found to result in increased levels of knowledge [32], few have reported long-term changes in sleep behaviour [27,32]. In fact, of all the nine sleep education programs undertaken in schools, only five resulted in behaviour change (minor improvements in sleep duration in two studies, improved sleep hygiene in three others).

One interpretation of this inconsistency is that increasing levels of sleep knowledge in children and/or their parents is insufficient to translate into behavior change. Unfortunately, surprisingly few of the most widely used sleep education programs report following an established and tested theoretical framework [32,35]. This lack of systematic theoretical foundations may account for the inconsistent success of sleep education in changing sleep behavior. However, even those who have utilised a behavioural change theoretical base, have not systematically seen last change in behavior patterns. Why?

## 3. Education to Behavioural Change

The challenge therefore is to understand and address what is missing in the current sleep education debate. The universal characteristics of behavioural change can be crucial to program design and must be applied to sleep education if risk prevention is to be achieved. Whilst previous sleep education programs and theories noted above address the interaction between individuals and their immediate environments, such as family and schools, what is missing from such models, are considerations of the impact of external influences and the broader social and environmental context of a child’s sleep. We propose that an integrative model guided by, but not replacing, existing theories could be what is needed for maximizing sleep behavior change. A model that acknowledges not only the inter- and intra-individual, family and community levels but also considers broader cultural, psychosocial, educational and political contexts.

### 3.1. Bronfenbrenner’s Ecological Systems Theory

One method by which this can be achieved is by applying the approach proposed by Bronfenbrenner’s Ecological Systems Theory.

The Ecological Systems Theory indicates and describes the surrounding structure of a child’s environment and its effect on the child’s development. While the immediate environment of a child is of crucial importance (e.g., home, family), the larger environment (e.g., school, community, society, education, political and cultural systems) is also recognised as being essential.

**Figure 2:**
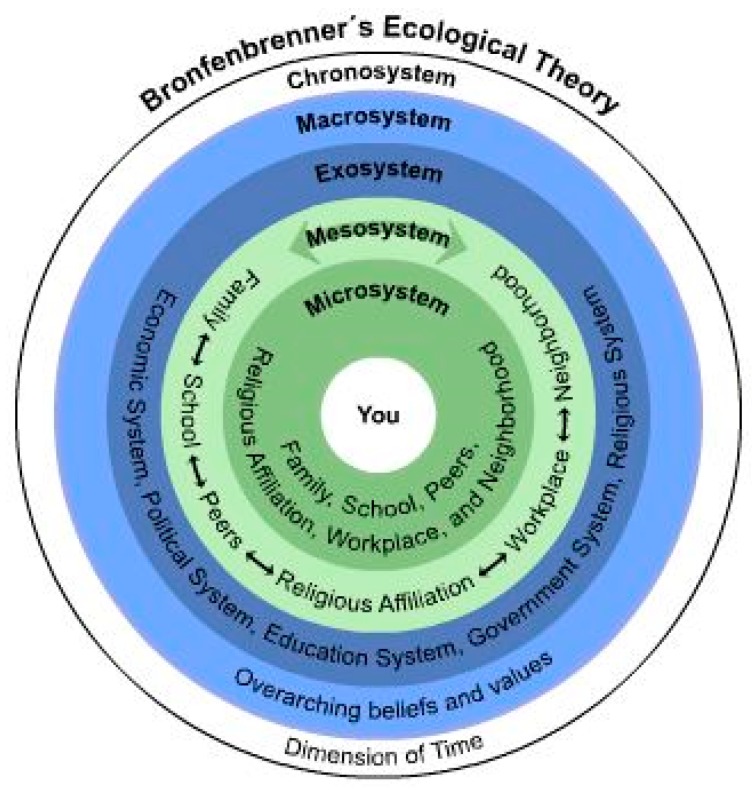
Bronfenbrenners Ecological Model. From Nielsen, J.N. (2011) [36].

Initially, Bronfenbrenner’s theory was comprised of the four well-known systems that impact development; Microsystem, Mesosystem, Exosystem and Macrosystem, with a fifth, the Chronosystem, taking into account the dimension of time related to the child’s development. Subsequently, Bronfenbrenner developed his theory into a more person-context model called the Bioecological Model (See Figure 2) [36]. Most sleep education programs have intuitively embraced elements of the Micro and Meso systems, but consideration of the Exo-, Macro- and Chronosystems adds an even broader perspective through which sleep education and awareness can be considered in a in preventative health model.

#### 3.1.1. The Micro and Meso Systems in Relation to Sleep

The first two of Bronfenbrenner’s systems, the Micro and Macrosystems, have been considered in some (not all) current sleep education programs and most current sleep education programs operate within those systems. In the microsystem there is focus on the child, family, school, religious institutions, neighbourhood and peers while the Mesosystem, focuses on the interrelationship between these components.

Clearly family and parents influence the sleep of young people, especially younger children. Parental influences such as guidelines for children’s good sleep hygiene practices (e.g., restricted television viewing, reduced night time food or drink consumption, calming activities before bed) and parental sleep knowledge have shown significant effects on children’s sleep practices and the capacity to change them [37,38,39]. Furthermore, studies have indicated large parental knowledge gaps regarding healthy sleep in children, and have shown that parents who are unaware of recommended sleep amounts were more likely to have children who did not obtain sufficient sleep [40]. Another study showed that parental knowledge and rule setting around bedtimes and media usage were positively related to improved sleep in adolescents [41].

These sleep hygiene recommendations are important, and knowledge about the importance of changing or improving sleep hygiene is strongly influenced by the family and peers at the Micro and Meso systems levels. Delivery of sleep education in schools is clearly not sufficient if we are to achieve sleep behaviour change, given that sleep and sleep hygiene practices occur within the family. Family inclusion in sleep education programs is paramount and some sleep education programs and several reviews [32,35] have identified that to including parents and families in school based programs (by conducting parenting information sessions and/or providing parents with information) is important. Interestingly, as children age however, the influence of peers becomes increasingly important with less parental jurisdiction and so the interrelationship between family and peers, as described in the Mesosystem, becomes important. This interplay between “significant others” such as family and peers, and how these groups view the importance awarded to good sleep is paramount to achieving behavior change. Sleep behavior change must be considered important, not just by the individual but by their family, their peers and their school community. This is one potential domain in which current sleep education programs could improve.

#### 3.1.2. The Exosystem: Economics and Education

Bronfenbrenner’s Exosystem identifies the roles of several wider systems that influence children’s development; two of which have direct relevance for a child’s sleep patterns and subsequently their sleep health. These two domains are (socio)-economic and education systems.

Firstly, socioeconomic status (SES) is an aspect of the wider economic environment that impacts each child’s sleep. In general, children of minority ethnicity and of lower socioeconomic status obtain less sleep and have more sleep difficulties than their counterparts [42]. This continues through adolescence, with Fredriksen and colleagues finding that adolescents who were more economically disadvantaged obtained less sleep [11]. Additionally, Crosby, LeBourgeois, and Harsh showed that in comparison to European, Americans and children from higher income backgrounds, low-income African American children were observed to have multiple parameters of poor sleep [43]. Whinery et al. report that those in the highest SES groups are significantly more likely to sleep well [44]. Expectations of improving sleep behavior through sleep education programs in lower SES households would need to consider this. SES is also directly related to the school system that a child attends (e.g., private vs. public education) and plausibly impacts the educational experiences for that child and the educational messages about sleep health. [44]. Would sleep education programs have to consider different and/or targeted methods of delivery in lower SES families and/or schools? Are parents in lower SES households and/or schools less able to engage with sleep education through their school community? Are the literacy levels of these parents taken into account when disseminating information to the students? How should be best engage families in lower SES situations to deliver sleep health messages that are relevant and modifiable for them? Sleep education needs to look further—not just at the individual, or school or peers, but rather the education system itself. Indeed, Moore et al. [42] propose a simple economic model that could be adapted to sleep, where health (sleep health) is partially determined by lifestyle (sleep hygiene and sleep behavior choices) and educational attainment (schooling and SES) which in turn depends on individual preferences, financial and employment status of families and time constraints. The interrelationship between the Micro, Meso and Exo systems here is apparent. In the absence of systematic sleep education in schools, inclusion of sleep education into school curricula depends then not only on the school itself and its capacity and willingness to include sleep health in curricula but also on school funding and resources. In Australia this funding is partially distributed by both state and federal governments, so sleep education is impacted by school and education system budgets, driven by policy and government decisions particularly in the public sector. Therefore, systematic sleep education requires organisational change at a political, policy and educational level. Furthermore, federally regulated national school curricula, certainly in Australia, may well be overstretched with content and sleep education has not yet found a permanent place amongst the common the health messages such as of diet and physical activity disseminated to children, families and communities. Without political and social acknowledgment of the importance of sleep and subsequent educational inclusion together it is unlikely that any sleep education program restricted to Meso and Micro levels will be successful in changing sleep behavior. We argue that of all the systems in Bronfenbrenner’s model, the Exosystem needs greater consideration in the discussion of preventative sleep education if we are to expect meaningful behaviour change.

#### 3.1.3. The Macrosystem: Culture

The Macrosystem influences the overarching beliefs and values upon a child, their family and their community, particularly in the domains of ethnicity, culture and social norms and highlights the inter relationship between them. Beliefs about the importance of sleep in different sub groups of our populations can and do define whether sleep is prioritised. This touches on the concept of the influence of ‘significant others’ discussed in social cognitive theory, where the opinions of important people in a child’s life significantly impact the success or otherwise of programs aimed at changing behaviour [45].

The Macrosystem has particularly interesting connotations in regards to sleep behaviour, as research has shown differences in sleep-related behaviours and attitudes across ethnicity and cultural practices in prioritising sleep practices and healthy sleep behaviours. For example, Huang and colleagues found that co-sleeping is very common in young children in China, with up to 53% of school-aged children in China engaging in co-sleeping [46]. However, the practice of co-sleeping, whilst widely debated as having advantages and disadvantages for children’s sleep [39], is much less common in modern westernised societies such as the United States of America, England and Australia and has even been promoted as negatively impacting the development of independence in young children [47]. These latter countries report that co-sleeping is contrary to individualist societies’ expectations of independence [48], so is less encouraged than in collectivist societies. Cultural or spiritual events may also have an effect on sleep patterning in children. For instance, in Balinese society, many spiritual observances and performances, which include both children and adults, occur at night and continue until daybreak or beyond, with sleep occurring during the course of the event as the individual’s needs or biological demands dictate [49]. Penman showed that cultural differences also exist in some Australian Aboriginal communities [50]. For example, the Yapa and Anangu people of Central Australia do not expect their children to fit into routines of eating and sleeping in particular places and at particular times, as is often expected in Anglo-Australian cultures [50]. Particular cultural or spiritual systems may therefore also alter how individuals or communities will conceptualise sleep and therefore how they conceptualise sleep problems. In addition, bedtime routines and bedtime are strongly influenced by culture. In many Latin and Asian cultures family meals are significantly later than those reported in English and many Australian families [51]. Those engaging in later meal and bedtimes are likely to receive less sleep due to consistent school start times. In English and Australian families late night meals may be avoided due mainly to the perceived need for children to be asleep at a culturally specified earlier bedtime. How should sleep messages cater for these differences? How much importance would families of these late night cultures place on sleep hygiene messages through school based programs if they do not align with their cultural practices? What does this mean for multi-cultural societies and sleep education in schools with a multi- ethic mix of children?

#### 3.1.4. The Chronosystem: Time

Finally, according to Bronfenbrenner’s model, the Chronosystem considers the overarching dimension of time and thus has the capacity to influence each of the other systems? Firstly, there have been secular changes in population level sleep duration and sleep knowledge over several decades [52]. A seminal study by Dollman et al. [53] showed approximately 1 h decrease in sleep duration between 1985 and 2005. It is believed this is largely due to the 24 h society and the increased use of technology which often displaces sleep. Sleep education messages need to keep abreast of societal changes and how they impact on sleep. Secondly sleep behaviour and the imperatives of changing sleep behaviour alter significantly over time as children age. For sleep education and sleep awareness, it is important to take into account that over their schooling, the exposure of children to sleep health messages will also change. No published studies have addressed sleep education in junior schools. Even if they did, sleep behaviour in a pre-school child is significantly different to a school-aged child and so the acquisition of sleep knowledge would be proportionate to need. Furthermore, whilst the need for sleep over the last decade has not diminished [54], the choices young people make about how they displace sleep for other activities (i.e., study, social activities) has changed, which in turn impacts and interrelates with the other systems in the model. Indeed, not one published sleep education study has shown sustained sleep behavior change over time even if and when changes were found immediately after education dissemination. Clearly future sleep education programs would do well to target sleep education programs to the age of the child but also these messages should not just be delivered once, but throughout the entire period of child development. This is true not only because repeated messages have been more successful in behavior change compared to singular disseminations [55] but also because different messages and different methods of dissemination are different for each developmental period. Indeed, as children age their relationship with their family, their school, their peers, and their community change and what they deem important changes with it. Persistent and continual sleep education messages across time and ages is important. So sleep education does indeed have a time/age dimension as encompassed by the Chronosystem, and the discourse will differ depending on whether it discusses junior school children or senior school adolescents. However, the basic understanding of the interaction between it and other systems according to Bronfenbrenner, remains the same.

In summary, the discussion above suggests that broadening our consideration of multiple societal, environmental and ecological factors as described by each of Brofenbrenner’s systems may add to the efficacy of sleep education programs? (See Table 1).

## 4. Limitations and Further Research

In this paper, we have attempted to demonstrate why current sleep education programs are not achieving long lasting changes in sleep health behaviour by highlighting what Bronfenbrenner considers important in behaviour change and applying this to children’s sleep. However, empirical research is required to evaluate our proposition. Other theories may apply equally as well or better, or indeed together, as part of a cohesive integration of the elements of many theories [56]. At the very least, different systems of the model may need to be emphasised or de-emphasised to address the requirements of different types of sleep problems, or to meet the needs of different age groups—including adults. The relationship between knowledge and action is particularly complex in relation to children’s health behaviours. Most current school sleep education models attempt to improve the knowledge of children, assuming that children are fully responsible and accountable for their behaviour. Applying Bronfenbrenner’s model suggests that there is a lot more to consider. Targeted sleep education programs that are able to take into account these factors interact with the broader environment may well have more success than those currently in usage. Furthermore, comparison of the outcomes of existing program design versus a program designed with the Ecological model principals in mind would be beneficial in evaluating the impact of broad based theory-informed and contextually relevant programs aimed at risk prevention. If broad based theoretically informed programs are found to be more effective, there will be a need to determine the best means of ‘retro-fitting’ theory into established programs, which may involve a degree of culture change as well as behaviour change. Recognising the potential benefits of integrating ronfenbrenner’s theory into existing behaviour change theories for sleep education programs may be an important step in our efforts to translate sleep education into sleep behavior change.

## Figures and Tables

**Table 1:** Brofenbrenner’s ecological systems approach to sleep education.

System Level	Domain	Sleep Impact	Solution
Individual child “you”	The individual child	• Individual differences in sleep need • Individual differences in the importance of sleep • Age • Gender • Temperamental differences in coping with sleep	• Deliver sleep education messages inclusive of individual differences • Promote overall message of sleep health and ability for consumers to critically assess individual sleep need.
Microsystem	Family Peers School Close community	• Family preferences • Parental influence and education • Peer pressure • School delivery of sleep education • Close community perceived importance of sleep	• Respect and address individual differences in families • Encourage a whole of school approach • Deliver community based messages of sleep health through close community organisations (sports clubs etc.)
Mesosystem	Relationship of Microsystem domains	• Family perceptions are influenced by: education, grandparent perceptions, peers and the importance placed upon sleep by schools, community and peers	• Recognise the relationships between all the above (one cannot be targeted without the other)
Exosystem	Education policy	• Sleep education delivered ad hoc in different schools with different agendas • Policy decisions by individual schools and education departments are not systematic • Little sleep education in medical curricula • School curricula are already overloaded	• Introduce systematic sleep education in conjunction with diet and physical activity in schools. • Develop policy guidelines for sleep health • Increase sleep education in medical curricula • Deliver sleep education from the Top down in medical training • Incorporate sleep into overall health messages across curricula areas and ages groups
	Economy	• Lower SES have poorer sleep • Poorer schools may not prioritise sleep due to curriculum pressures	• Targeting low SES households and/or schools, where sleep health is poorest
Macro System	Culture and ethnicity	• Different cultures prioritise sleep in different ways	• Culturally sensitive sleep education inclusive of how best to improve sleep practices and accounting for cultural differences
Chronosystem	Time	• Sleep changes with age • Sleep priorities change with age • One off message are unlikely to have long lasting impact.	• Target specific sleep education to each age group. • Consider that one off messages are not sufficient and sleep health promotion messages needs to be across years and ages
